# Maternal age and risk of early neonatal mortality: a national cohort study

**DOI:** 10.1038/s41598-021-80968-4

**Published:** 2021-01-12

**Authors:** Yoo-Na Kim, Dong-Woo Choi, Dong Seop Kim, Eun-Cheol Park, Ja-Young Kwon

**Affiliations:** 1 Department of Obstetrics and Gynecology, Institute of Women’s Medical Life Science, Yonsei University College of Medicine, Yonsei University Health System, Seoul, Republic of Korea; 2 Department of Public Health, Graduate School, Yonsei University, Seoul, Republic of Korea; 3 Institute of Health Services Research, Yonsei University, Seoul, Republic of Korea; 4 Department of Medicine, Graduate School, CHA University, Gyeonggi-do, Republic of Korea; 5 Department of Preventive Medicine, Yonsei University College of Medicine, Seoul, Republic of Korea

**Keywords:** Risk factors, Pregnancy outcome

## Abstract

Advanced maternal age (AMA) is a growing trend world-wide and is traditionally defined as childbearing in women over 35 years of age. The purpose of our study was to determine the maternal age group within the Korean population, in which the risk of early neonatal mortality is increased. Korean birth and mortality data from 2011 to 2015 were used to estimate the influence of maternal age on the risk of early neonatal mortality. A Poisson regression was used for the analysis of multiple clinical variables such as year of delivery, maternal age, gestational age, infant gender, birth weight, multiple birth, parity, and socioeconomic variables. Furthermore, a generalized additive model was used to determine the maternal age at which the risk for neonatal mortality increases. We included 2,161,908 participants and found that 49.4% of mothers were 30–34 years of age at delivery. The proportion of mothers aged 35 and above increased over the 5-year analysis period. A maternal age lower than 29 years or higher than 40 years was associated with a relatively higher risk of early neonatal mortality. The trend and magnitude of the age-related risk on early neonatal mortality were independent of maternal socioeconomic factors such as living in an obstetrically underserved area, education level, and employment status. Furthermore, we showed that the risk for early neonatal mortality was higher until the maternal age of 28. However, there were no significant changes in the risk between the age of 35 and 40 years. According to recent national-wide data, age-related risk for early neonatal mortality is only apparent for mothers ≥ 40 years old whereas, age between 35 and 39 are not at increased risk for early neonatal mortality, despite being classified as AMA.

## Introduction

Changes in social habits such as late marriage, widespread use of contraception, and accessibility to infertility treatments have contributed to a world-wide increase in child-bearing age^1,2^. This is more noticeable in rapidly aging societies, due to an upwards shift in the age distribution of fertile women. According to South Korean national statistics, the median age at first childbirth has increased from 29.8 to 32.2 years from 2009 to 2019^3^. The percentage of advanced maternal age (AMA) mothers, traditionally defined as childbearing women aged 35 years and older, has increased from 15.4% in 2009 to 33.4% in 2019. Similarly, in the United States, the percentage of women who had their first child in the 35–39 age bracket increased six-fold from 1973 to 2006^4^, and the proportion of mothers giving birth at > 40 of age continues to rise in 2018^5^. This rise in maternal age in both developing and developed nations led to the appearance of the “very advanced maternal age” category, for childbirths occurring between 45 and 50 years of age^6–9^.

Age cut-off of 35 to define AMA was based on studies on age-related adverse pregnancy outcome^10^, where outcome was a composite of maternal^11,12^, fetal^13,14^, and perinatal outcomes^15–18^. The proportion of AMA in these relatively older studies was small compared to the more contemporary cohort. Therefore, it was difficult for the clinicians to pinpoint an exact age cut-off at which exclusively the neonatal risk increases when counseling AMA. Moreover, advancement in healthcare should be taken into account when assessing maternal age-related neonatal risk. Therefore, we conducted a population-based retrospective cohort study of births in South Korea from 2011 to 2015 to investigate maternal age-related early neonatal mortality outcome.

## Materials and methods

### Data source

**Figure 1:**
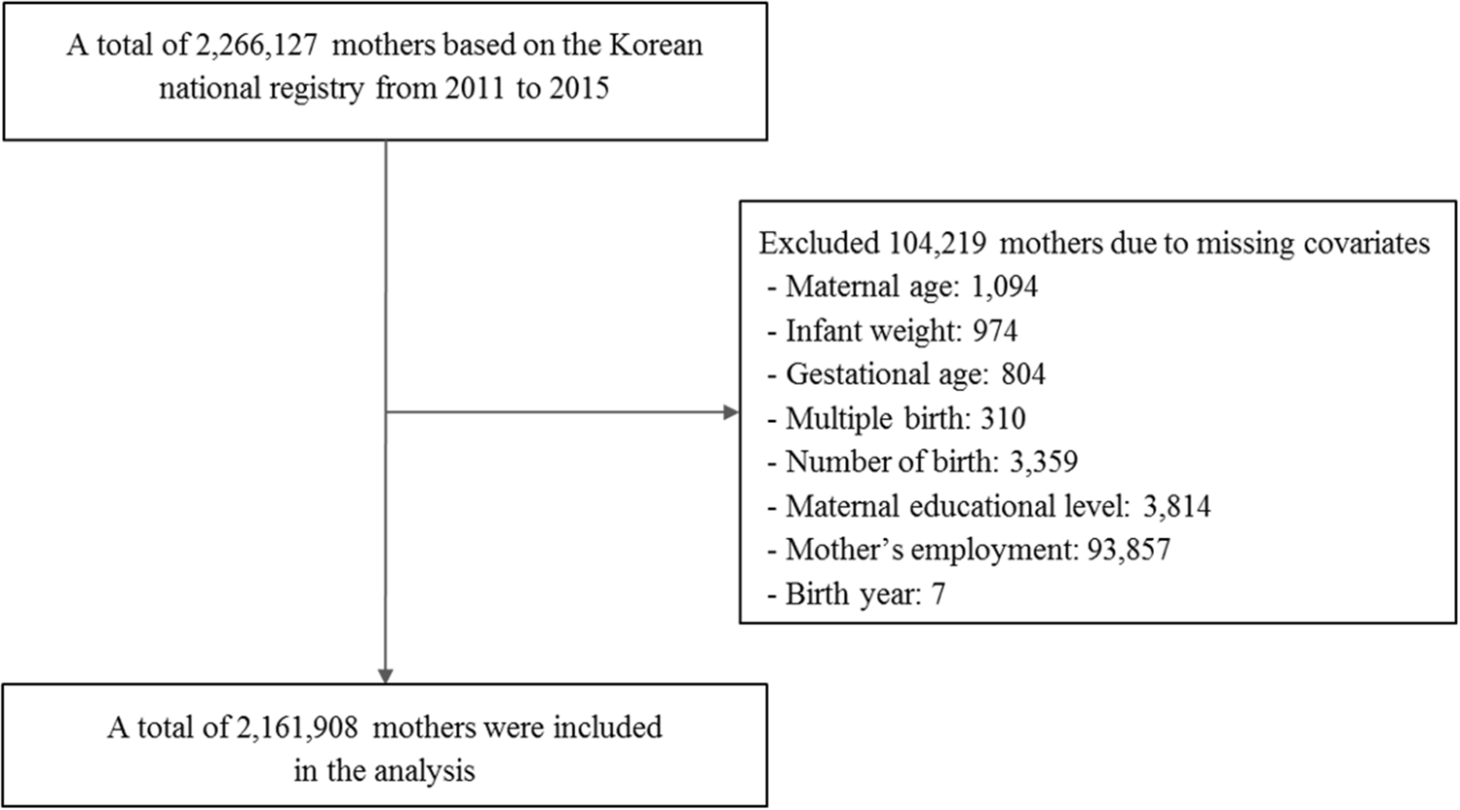
Flow diagram of the study population.

Data regarding fetal, neonatal, infant mortality, and live births from 2011 to 2015 were obtained from the ‘Korean Vital Statistics,’ through the KOrean Statistical Information Service (KOSIS, https://kosis.kr). Korean Vital Statistics is a national database containing information about childbirth, death, marriage, and divorce rates in Korea, complemented by both medical and socioeconomic information. Therefore, information on maternal age and corresponding socioeconomic details were extracted alongside neonatal birth details (such as gestational age, birth weight, region of birth, occurrence of multiple births, and year of birth). From a total of 2,266,127 participants registered in KOSIS from 2011 to 2015, we included 2,161,908 participants registered in in the final analysis (Fig. 1), after excluding those with missing covariates such as birth information and maternal information. The present study was approved by the Yonsei University Severance Hospital institutional review board (4-2019-1181) and informed consent was waived by the Ethics committee as The Korean Vital Statistics database consists of public, anonymized, and unidentified patient data. The research was performed in accordance with relevant guidelines/regulations.

### Variables

Maternal age was considered as an independent variable and divided into six distinct categories of age groups (“≤ 19”, “20–24”, “25–29”, “30–34”, “35–39”, and “ ≥ 40”). The primary outcome was early neonatal mortality (ENM), defined as death within the first 28 days after birth. Covariates included infant gender, size for gestational age (“small for gestational age", "appropriate for gestational age", and "large for gestational age"), infant weight (“≤ 1499 g”, “1500–2499 g”, “2500–3499 g”, and “ ≥ 3500 g”), gestational age (“≤ 28^+0 ^weeks”, “28^+1^–32^+6^ weeks”, “33^+0^–36^+6^ weeks”, and “ ≥ 37^+0 ^weeks”), multiple birth (“single” and “twins or more”), parity (0, 1, ≥ 2), maternal education level (“high school or under” and “university or above”), maternal employment status (“employed” and “not employed”), and year of delivery. The size for gestational age were categorized based on the percentile distribution of weight for a given gestational age at delivery (small for gestational age defined as below 10 percent percentile; large for gestational age defined as above 90 percent percentile)^19^. The location of delivery was rated as obstetrically underserved (“yes” and “no”) according to a previous report^20^.

### Statistical analysis

A chi-squared test was used to compare the descriptive statistics of baseline characteristics. The risk ratio for ENM was estimated by a Poisson regression with covariates. After then, we conducted a generalized additive model (GAM), which consists of a combination of generalized linear and additive models^21,22^, to investigate the non-linear effects on maternal ages and to determine the maternal age at which the risk for ENM starts increasing by the optimized smooth functions. Through a link function to capture the association between the expectation of explained variables and nonparametric explanatory variables^23^, the GAM was previously shown to be superior at analyzing complex nonlinear relationships^24^. In the analysis, the GAM was fitted with a binomial error distribution and a logit link function, with spline smoothing. After comparing the smoothing term from 3 to 8, 3 was chosen as the effective degrees of freedom for the smoothing parameter which was the smallest Akaike’s Information Criterion (AIC) and Bayesian Information Criterion (BIC) (sTable 1). The smoothing parameter estimate for P-value for maternal age were significant (*P* = 0.0059). Subsequently, subgroup analysis was performed according to obstetrically underserved areas, maternal educational level, and year of delivery to investigate the heterogeneity of effect sizes for the association between maternal age and risk of ENM after controlling other covariates. SAS version 9.4 (SAS institute, Inc., Cary, NC, USA) was used for all statistical analyses. Statistical significance was defined as *P* < 0.05.

## Results

### Demographics of participants

The characteristics of the study participants are presented in Table 1. The maternal age groups were distributed as follows: 0.58% (n = 12,613) for under 19, 5.01% (n = 108,254) for 20–24, 24.61% (n = 531,982) for 25–29, 49.39% (n = 1,067,834) for 30–34, 17.88% (n = 386,598) for 35–39, and 2.53% (n = 54,627) for over 40 years of age. According to the maternal age groups, numbers of early neonatal deaths were distributed as follows: 0.12% for under 19 (n = 15), 0.06% for 20–24 (n = 65), 0.04% for 25–29 (n = 230), 0.05% for 30–34 (n = 566), 0.06% for 35–39 (n = 241), and 0.11% for over 40 years of age (n = 60).

**Table 1:** Characteristics of the study population.

Variables	Total birth	Early neonatal mortality		*p* value
		N	%	
**Maternal age (years)**				< 0.0001
≤ 19	12,613	15	0.12	
20–24	108,254	65	0.06	
25–29	531,982	230	0.04	
30–34	1,067,834	566	0.05	
35–39	386,598	241	0.06	
≥ 40	54,627	60	0.11	
**Infant gender**				0.0019
Male	1,109,672	658	0.06	
Female	1,052,236	519	0.05	
**Size for gestational age**				< 0.0001
Small for gestational age	136,655	254	0.19	
Appropriate for gestational age	1,784,722	829	0.05	
Large for gestational age	240,531	94	0.04	
**Infant weight**				< 0.0001
≤ 1499 g	13,727	725	5.28	
1500–2499 g	105,126	183	0.17	
2500–3499 g	1,491,070	219	0.01	
3500 g +	551,985	50	0.01	
**Gestational age**				< 0.0001
≤ 28^+0 ^weeks	5182	562	10.85	
28^+1^–32^+6^ weeks	16,656	213	1.28	
33^+0^–36^+6^ weeks	117,435	159	0.14	
≥ 37^+0 ^weeks	2,022,635	243	0.01	
**Multiple birth**				< 0.0001
Singles	2,089,608	880	0.04	
Twins or more	72,300	297	0.41	
**Parity**				< 0.0001
0	1,109,080	561	0.05	
1	827,957	440	0.05	
≥ 2	224,871	176	0.74	
**Obstetrically underserved areas**				0.0022
Yes	620,910	386	0.06	
No	1,540,998	791	0.05	
**Maternal educational level**				< 0.0001
High school or lower	592,024	400	0.07	
University or above	1,569,884	777	0.05	
**Maternal employment status**				0.0010
Employed	713,359	335	0.05	
Unemployed	1,448,549	842	0.06	
**Year of delivery**				0.9650
2011	466,077	253	0.05	
2012	461,092	247	0.05	
2013	413,781	235	0.06	
2014	410,690	219	0.05	
2015	410,268	223	0.05	
**Total**	2,161,908	1177	0.05	

### Age-related risk analysis

The risk ratios for ENM are shown in Table 2. Using the maternal age group of 30–34 as reference, higher risk ratios were found for a maternal age of 19 years or below (RR: 1.84, 95% CI: 1.77–1.91), 20–24 (RR: 1.39, 95% CI: 1.37–1.42), 25–29 years (RR: 1.02, 95% CI: 1.01–1.03), and 40 years or above (RR: 1.26, 95% CI: 1.23–1.28). The risk ratio for ENM was not increased in 35–39 years of age (RR: 0.89, 95% CI: 0.88–0.90).

**Table 2:** Risk ratios (RR) (95% CI) for early neonatal mortality according to factors.

Variables	Early neonatal mortality		
	RR	95% CI	
**Maternal age (years)**			
≤ 19	1.84	1.77	1.91
20–24	1.39	1.37	1.42
25–29	1.02	1.01	1.03
30–34	1.00		
35–39	0.89	0.88	0.90
≥ 40	1.26	1.23	1.28
**Infant gender**			
Male	1.17	1.16	1.18
Female	1.00		
**Size for gestational age**			
Small for gestational age	1.94	1.92	1.96
Appropriate for gestational age	1.00		
Large for gestational age	2.07	2.03	2.10
**Infant weight**			
≤ 1499 g	14.28	13.78	14.81
1500–2499 g	4.91	4.76	5.05
2500–3499 g	1.88	1.84	1.93
3500 g +	1.00		
**Gestational age**			
≤ 28^+0 ^weeks	109.57	106.60	112.63
28^+1^–32^+6^ weeks	19.77	19.29	20.27
33^+0^–36^+6^ weeks	5.44	5.34	5.54
≥ 37^+0 ^weeks	1.00		
**Multiple birth**			
Singles	1.00		
Twins or more	1.04	1.03	1.05
**Parity**			
0	1.00		
1	0.96	0.95	0.97
≥ 2	1.18	1.17	1.20
**Obstetrically underserved area**			
Yes	1.13	1.12	1.14
No	1.00		
**Maternal educational level**			
High school	1.00	0.99	1.01
University or above	1.00		
**Maternal employment status**			
Employed	0.97	0.96	0.98
Unemployed	1.00		
**Year of delivery**			
2011	1.00		
2012	0.95	0.94	0.96
2013	0.98	0.97	0.99
2014	0.93	0.92	0.95
2015	0.92	0.91	0.93

**Figure 2:**
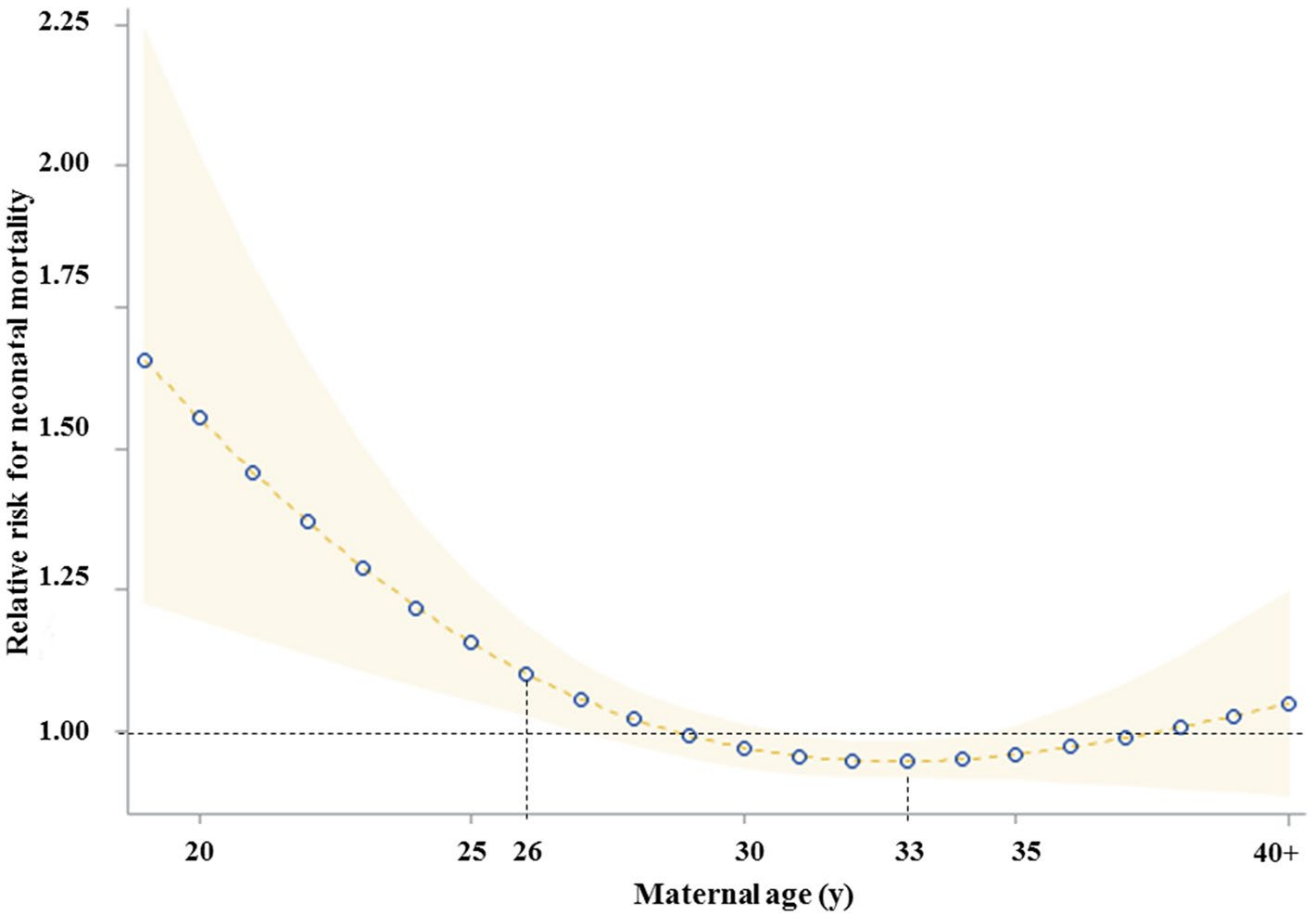
Association between maternal age and early neonatal mortality.

GAM analysis showed a reverse J-shaped association between maternal age and ENM (Fig. 2). According to the age segmentation, the trough was shown for a maternal age of 33 years (RR: 0.94, 95% CI: 0.91– 0.98). Maternal age from 28 years (RR: 1.02, 95% CI: 1.02– 1.18) to 19 or below (RR: 1.66, 95% CI: 1.23– 2.25) had a high-risk ratio for ENM compared to other maternal age groups. However, there was no association between maternal age and ENM from 35 to 39 years.

### Subgroup analysis

A subgroup analysis showed that, for a maternal age of 35–39 years, neither of the analyzed subgroups such as obstetrically underserved area, maternal educational level, and maternal employment was heterogeneously associated with an increased risk of ENM when compared to a maternal age of 30–34 years (Table 3). The risk ratio for 35–39 year-old mothers who delivered in an obstetrically underserved area was 0.92 (95% CI: 0.90–0.94) and 0.89 (95% CI: 0.88–0.90) for those who delivered in an obstetrically well-equipped region. For the same age group, the risk ratio was 0.94 (95% CI: 0.92–0.96) for those who had a high school (or lower) education and 0.87 (95% CI: 0.86–0.88) for those with university (or higher) education. Across the span of 5 years, mothers aged 35–39 years did not have a higher risk ratio for ENM than mothers aged 30–34 years; in fact, the risk ratio for mothers aged 35–39 years showed a trend to be lower than the one for the 30–34 year age bracket. However, other maternal age groups were strongly associated with increasing ENM.

**Table 3:** Risk ratios (RR) (95% CI) for neonatal mortality according to subgroups.

Subgroups	Maternal age	Early neonatal mortality		
		RR	95% CI	
**Obstetrically underserved area**				
Yes	≤ 19	2.53	2.41	2.66
	20–24	1.64	1.60	1.69
	25–29	1.00	0.98	1.01
	30–34	1.00		
	35–39	0.92	0.90	0.93
	≥ 40	1.21	1.16	1.25
No	≤ 19	1.26	1.19	1.33
	20–24	1.21	1.18	1.24
	25–29	1.03	1.01	1.04
	30–34	1.00		
	35–39	0.89	0.88	0.90
	≥ 40	1.27	1.24	1.30
**Maternal educational level**				
High school or lower	≤ 19	1.76	1.69	1.83
	20–24	1.36	1.32	1.40
	25–29	1.19	1.17	1.22
	30–34	1.00		
	35–39	0.94	0.92	0.96
	≥ 40	1.03	1.00	1.07
University or above	≤ 19			
	20–24	1.39	1.35	1.43
	25–29	0.95	0.94	0.96
	30–34	1.00		
	35–39	0.87	0.86	0.88
	≥ 40	1.53	1.49	1.56
**Year of delivery**				
2011	≤ 19	1.60	1.47	1.73
	20–24	1.61	1.54	1.67
	25–29	1.10	1.07	1.12
	30–34	1.00		
	35–39	0.98	0.95	1.00
	≥ 40	1.41	1.35	1.47
2012	≤ 19	2.65	2.49	2.83
	20–24	1.70	1.64	1.76
	25–29	1.35	1.32	1.37
	30–34	1.00		
	35–39	0.93	0.91	0.95
	≥ 40	1.64	1.59	1.70
2013	≤ 19	1.50	1.38	1.64
	20–24	1.02	0.97	1.07
	25–29	0.84	0.82	0.86
	30–34	1.00		
	35–39	0.91	0.89	0.93
	≥ 40	1.25	1.20	1.31
2014	≤ 19	2.33	2.17	2.51
	20–24	1.49	1.43	1.55
	25–29	0.74	0.72	0.76
	30–34	1.00		
	35–39	0.79	0.77	0.81
	≥ 40	1.15	1.10	1.20
2015	≤ 19	–		
	20–24	1.15	1.10	1.21
	25–29	1.10	1.07	1.12
	30–34	1.00		
	35–39	0.91	0.88	0.93
	≥ 40	0.81	0.77	0.85

## Discussion

According to our review of over 2 million mothers, 49.4% of all deliveries fall into the category of maternal age between 30 and 34 years. Moreover, mothers in the age range of 35 to 39 years old accounted for as much as 17.9% overall, increasing from 15.7% in 2011 to 21.1% in 2015, indicating that the age of motherhood is rapidly increased in South Korea. The proportion of mothers 40 years or older also appears to be increasing as 2.24% and 2.85% of the total childbearing mothers fell into this age category in 2011 and 2015, respectively. A world-wide demographic shift has fomented studies on the adverse impact of a very advanced maternal age in mothers aged 40 to 45 years (or even older)^1^. The current increase in childbearing women aged 35–39 or more has raised questions regarding if this age range should be considered “high-risk”. Analysis of national data by a Poisson regression analysis showed that ENM rate was not increased in mothers aged 35–39 years old. In fact, this age category even had a slightly lower rate of relative risk (0.9) compared to the reference group of mothers aged 30–34 years old. The U-shaped distribution reveals an increased risk of ENM at both extremes of the age ranges, suggesting that the age-related ENM risk increases in a continuous^25,26^, rather than step-wise fashion as methodological approaches from previous studies had suggested. Therefore, our results suggest a multifaceted approach to define AMA in today’s society. Ideally, this should help spare both physicians and patients from ungrounded fears related to ENM, which has been shown to lead to unreasonably low thresholds for obstetric intervention and primary cesarean section^27,28^.

Our data suggested that Korean mothers are not only getting older, but are also more educated and involved in workforce at time of delivery. Previous studies have suggested that the maternal education level and employment status may influence not only the outcome of the pregnancy, but also child rearing^29^. Older mothers did not demonstrate reduced parenting skills^30^; in fact, children of older mothers may even be at an advantage in terms of nutritional status and education^31^. Similarly, our subgroup analysis suggested that maternal education and employment status—factors that are associated with increased maternal age—were protective for ENM. Demographically, the proportion of mothers with a university (or higher) education increased from 65% in 2011 to 77% in 2015; the proportion of mothers who were employed at time of delivery increased from 29.8% in 2011 to 36.8% in 2015. If the shift in maternal age is accompanied by changes in the educational and employment status of the mothers, the age-related adverse effect could be further moderated. Older mothers who are educated and employed may be socioeconomically more stable than their younger counterparts, as data showed that older mothers were less likely to deliver in obstetrically underserved areas (25.65% for 35–39 years and 36.96% for 20–24 years) and this may have contributed in preventing ENM in 35–39 years group.

The place of delivery was a significant determinant of ENM when potential factors were taken into consideration. Consistently throughout the years, approximately one fourth of the mothers delivered in an obstetrically underserved district. Older mothers having a higher prevalence of age-related diseases, such as hypertension and diabetes, would be the primary beneficiaries of improved antenatal care and public health interventions in currently obstetrically underserved areas. From the aspect of antenatal care, previous studies have suggested that certain controllable risk factors such as smoking and obesity can be as critical as biological processes related to aging^32,33^. From a clinical aspect, ENM may be influenced by the availability of neonatal resuscitation techniques and quality of neonatal intensive care unit facilities. Therefore, the outcome of any study on maternal or neonatal mortality would be highly sensitive to the setting in which the study was conducted, nation-wise or in terms of access to healthcare^34,35^. In developing countries, access to care and appropriate education of early danger signs of high risk pregnancy are likely to reduce neonatal deaths^36^. In high-income countries, survival of neonates born may be improved by centralizing at-risk deliveries to well-equipped centers where multidisciplinary teams are available around the clock^37^.

South Korea has the highest mean maternal age among all OCED countries^1^ and has an ethnically homogenous cohort backed by the nationalized health care system, making it an ideal country to assess a rare adverse pregnancy outcome such as ENM. An interesting observation was that while the incidence of congenital malformation and very low birthweight (< 1500 g) births increased alongside with the proportion of AMAs^38^, the national maternal and infant mortality rate further decreased during the study period (KOSIS database). In retrospect, concurrent to the study period, two healthcare policies were introduced to give direct financial support to high-risk mothers and to reduce the regional disparities in antenatal and delivery services^39^. Thus, national wide measures such as targeted policy intervention and logistic improvements may have helped reduce ENM in the advanced maternal age of 35–39.

Although ENM is a composite outcome of multiple clinical factors, we were not able to directly capture factors driving the patterns observed in this cohort as it was based on national database with limited information. However, maternal age-related factors that are known to be associated with perinatal death, such as congenital birth defect^14^, preterm birth^18^, and preeclampsia^26^, and these factors are likely to be more severe in each category for > 40 years^7,17^. Thus, it can be carefully speculated that the reason ENM remained still high in the mothers over 40 years of age despite the degree of social support, may be accountable to congenital anomalies, chromosomal abnormality and extremely preterm or very low birth weight complicating neonates.

Lastly, the statistical distribution of the ENM outcomes in both extremes of the age group should be interpreted with caution because of relatively small number of events. Nevertheless, two additional observations can be made. First, higher risk of ENM was shown in the extremely young age group. Based on our data, the increased age-related risk of ENM was consistently observed in those < 19 in comparison to those between 20 and 25 of age. Such observation is in agreement with previous reports on the adverse pregnancy outcomes in the young age group especially in the context of teenage pregnancy^40^. Second, the increased variability in the outcomes of the extremely old patient who are above 40 years old. Even taking into consideration the influence of relatively small sample size, still it is possible that the outcomes in this very elderly cohort are more vulnerable to the changes in the modifiable, socioeconomic factors^36,41^. For age group with volatile pregnancy outcome, additional protective social measures and careful pregnancy counselling may be considered for the age group with volatile pregnancy outcome; not based on a strict age-defined cut-off of 35 or older.

Our study has several limitations. First, since our observational study draws from a national registry, certain clinical details could not be obtained. For instance, neonatal and maternal complications could not be controlled because details such as the rates of neonatal intensive care unit admission, the length of the interpregnancy interval, maternal thrombosis, and postpartum hemorrhagic were not included in the analysis. Moreover, stillbirth data are lacking, and other socioeconomic measures including body mass index and smoking were not taken into consideration. Second, due to the retrospective nature of the study, a causal relationship could not be established. Third, although we observed a trend in the association between maternal age and ENM, the time span of data collection studied was relatively short for us to observe a significant change over time.

In conclusion, based on the analysis of recent national-wide data, age-related risk for early neonatal mortality was apparent for mothers of 40 years of age. Furthermore, the impact of a maternal age-shift on ENM was analyzed to show that the outcomes of mothers aged 35–39 years were comparable to those at 25–34 years of age, despite being classified as AMA according to traditional definition. These findings suggest public health efforts should be taken to improve care for reproductive age of over 40 in order to reduce ENM along with other associated controllable risk factor such as delivering in obstetrically underserved areas.

## Supplementary Information

Supplementary Table 1.
