# Supplementary material for: Maternal age and risk of early neonatal mortality: a national cohort study
Source: Sci Rep. 2021 Jan 12;11:814. doi: 10.1038/s41598-021-80968-4 (PMC7804272; doi:10.1038/s41598-021-80968-4)
Supplement: Supplementary file 1 — Supplementary Table 1. [file 41598_2021_80968_MOESM1_ESM.docx]

**Title: Maternal age and risk of early neonatal mortality: a national cohort study**

**Short title: Maternal age and early neonatal mortality**

**Yoo-Na Kim** **^1¶^, Dong-Woo Choi ^2,3¶^, Dong Seop Kim^4^, Eun-Cheol Park^3,5^* Ja-Young Kwon^1^***

^1^ Department of Obstetrics and Gynecology, Institute of Women's Medical Life Science, Yonsei University College of Medicine, Yonsei University Health System, Seoul, Korea

^2^ Department of Public Health, Graduate School, Yonsei University, Seoul, Republic of Korea

^3^ Institute of Health Services Research, Yonsei University, Seoul, Republic of Korea

^4^ Department of Medicine, Graduate School, CHA University, Gyeonggi-do, Republic of Korea

^5^ Department of Preventive Medicine, Yonsei University College of Medicine, Seoul, Republic of Korea

*** Co-corresponding authors**

E-mail: [jaykwon@yuhs.ac](mailto:jaykwon@yuhs.ac) (JYK)

E-mail: [ecpark@yuhs.ac](mailto:ecpark@yuhs.ac) (ECP)

**^¶^These authors contributed equally to this work.**

**Keywords:** advanced maternal age, pregnancy, delivery, early neonatal mortality.

| sTable 1. Results of fit statistics and tests for smoothing componentss according to effective degrees of freedom | | | | | | |
| --- | --- | --- | --- | --- | --- | --- |
| Fit statistics |  |  |  |  |  |  |
| Effective degrees of freedom | 21 | 22 | 23 | 24 | 25 | 26 |
| AIC | 12,942 | 12,943 | 12,942 | 12,944 | 12,944 | 12,944 |
| BIC | 13,206 | 13,220 | 13,231 | 13,246 | 13,259 | 13,271 |
| Tests for Smoothing Components |  |  |  |  |  |  |
| Effective degrees of freedom | 3 | 4 | 5 | 6 | 7 | 8 |
| Chi-square | 10.2746 | 11.1085 | 15.5850 | 15.6023 | 16.4833 | 19.5978 |
| P-value | 0.0059 | 0.0112 | 0.0036 | 0.0081 | 0.0114 | 0.0065 |
